# Impact of Carotid Endarterectomy on Choroidal Thickness and Volume in Enhanced Depth Optical Coherence Tomography Imaging

**DOI:** 10.1155/2020/8326207

**Published:** 2020-03-02

**Authors:** Elżbieta Krytkowska, Monika Masiuk, Miłosz P. Kawa, Aleksandra Grabowicz, Paweł Rynio, Arkadiusz Kazimierczak, Krzysztof Safranow, Piotr Gutowski, Anna Machalińska

**Affiliations:** ^1^First Department of Ophthalmology, Pomeranian Medical University, Szczecin, Poland; ^2^Department of General Pathology, Pomeranian Medical University, Szczecin, Poland; ^3^Department of Vascular Surgery and Angiology, Pomeranian Medical University, Szczecin, Poland; ^4^Department of Biochemistry and Medical Chemistry, Pomeranian Medical University, Szczecin, Poland

## Abstract

**Purpose:**

Carotid endarterectomy (CEA) is considered an effective therapeutic method for improving ocular circulation. The choroid is a predominantly vascular tissue; thus, systemic and local vascular alterations may influence its morphology and function. The aim of the current study was to analyse changes in choroidal thickness and volume in patients with significant internal carotid artery stenosis (ICAS) before and after unilateral CEA.

**Methods:**

The 42 eyes of the 21 asymptomatic patients included in the study were divided into two groups: those ipsilateral (EIE) and those contralateral (ECE) to CEA. All participants underwent a complete ophthalmologic examination, including enhanced depth imaging-optical coherence tomography (EDI-OCT). A comparative analysis of subfoveal thickness (CT) and choroidal volume (CV) measured in nine Early Treatment of Diabetic Retinopathy Study (ETDRS) subfields at baseline, on the 2nd day after CEA, and in the 3rd month after CEA was performed.

**Results:**

In the EIE and ECE groups, no significant differences in either CT or CV values before and on the 2nd day after the CEA were observed. In the EIE group, a significant increase in CT and CV in the 3rd month after CEA compared to baseline was noted in the specific ETDRS region. Changes in CT and CV after surgery were positively correlated with the participants' physical activity status and diastolic blood pressure and negatively correlated with the participants' age and smoking status. Additionally, the analysis of changes in CV after CEA showed a positive correlation between the EIE and ECE groups.

**Conclusions:**

CT and CV fluctuations in the central and perifoveal areas visualized with EDI-OCT enabled the observation of the processes of tissue adaptation to variable blood flow conditions.

## 1. Introduction

The choroid is one of the most vascularized tissues of the body [1]. Its main function is supplying oxygen and nutrients to the outer retina, particularly in the foveal avascular zone, where the choroid is the exclusive blood source for the retina [2, 3]. The choroid is considered to have additional functions, such as thermoregulation and secretion of growth factors [1, 2]. Additionally, the choroid plays an important role in the regulation of intraocular pressure (IOP) due to its control of aqueous humour outflow regulation [1].

Localization between the overlying pigmented retinal epithelium (RPE) and the dense, opaque sclera beneath makes the choroid difficult to visualize. Conventional optical coherence tomography (OCT) is affected by the blocking effects of melanin and the scattering properties of the blood vessels. The introduction of modern OCT modalities, including enhanced depth imaging (EDI) or swept source (SS) OCT, has led to improvements in choroid imaging [4]. In recent years, there has been a growing interest in choroid analysis due to its potential role in the pathophysiology of many vision-threatening ocular disorders, including age-related macular degeneration (AMD), central serous chorioretinopathy, and degenerative myopia [5–8]. Studies have shown that choroidal thickness (CT) is a reliable and sensitive indicator of changes in many physiological and pathological conditions of the choroid [6, 9–12]. According to studies by Kim et al., subfoveal CT may be a clinically valuable indirect indicator of subfoveal ocular perfusion status because mean ocular perfusion pressure was significantly associated with subfoveal CT in eyes with <6 dioptres of myopia [13].

The choroid is predominantly composed of blood vessels organized in the choriocapillaris, Sattler's and Haller's layers [1, 2]. The blood supply to the choroid arises from the internal carotid artery (ICA) via the ophthalmic and posterior ciliary arteries (PCAs) [2]. It is currently believed that vascular disorders are the basis of many ocular diseases, including AMD, glaucoma, and diabetic retinopathy [14–16]. Insufficient blood supply to the eye due to internal carotid artery stenosis (ICAS) may manifest as acute or chronic ischaemic symptoms [17–20]. Carotid endarterectomy (CEA) is considered an effective therapeutic method for improving cerebral and ocular circulation [21–23]. Moreover, improvements in blood flow within the ophthalmic, central retinal, and posterior ciliary arteries as well as in the retinal and choroidal circulation have been documented [21, 22, 24–26].

There are few studies evaluating choroidal thickness measured with OCT in the context of carotid artery stenosis, and only two have assessed alterations in choroidal thickness after carotid endarterectomy. In addition, the conclusions from these two works do not overlap [27, 28]. Both are based mainly on a single CT measurement in the subfoveal region that was performed manually by the authors. This methodology does not cover all choroid parameters and can lead to biased results. An analysis of volume based on central 6 mm perifoveal B-scans of the choroid could provide more precise information on the impact of CEA on choroidal parameters.

Thus, the aim of our study was a comprehensive analysis of the changes in choroidal thickness and volume in patients with significant ICAS before and after unilateral carotid endarterectomy. To our knowledge, this is the first report analysing choroidal volume (CV) changes after CEA.

## 2. Materials and Methods

### 2.1. Criteria for Patient Inclusion and Assessment

Initially, 21 patients (42 eyes) with significant internal carotid artery stenosis who were subjected to unilateral endarterectomy were recruited to the study. The ophthalmological inclusion criterion was a best-corrected distance visual acuity (BCDVA) >0.8 (on the Snellen chart). The exclusion criteria included ischaemic ocular syndrome and other ophthalmic and systemic conditions that may affect the choroidal thickness, such as AMD, high refractive errors (over ±4 dioptres of spherical equivalent), glaucoma, pachychoroid spectrum disorders (including central choroid chorioretinopathy and polypoidal choroidal vasculopathy), choroiditis, retinopathy (any type), vitreomacular changes (such as an epiretinal membrane), a history of retinal detachment, or serious ocular trauma. Eyes were excluded if they had cataract surgery in the previous 6 months, any previous laser therapy, vitreoretinal surgery, or a history of intraocular injections.

### 2.2. Ophthalmologic Characteristics

All participants underwent a complete ophthalmologic examination, including the following: best-corrected distance visual acuity with Snellen charts, slit lamp biomicroscopy and a detailed fundus examination after pupil mydriasis with 1% tropicamide solution, IOP measurement with a Goldman applanation tonometer (Zeiss AT 030, Carl Zeiss, Jena, Germany), and axial length (AXL) and anterior chamber depth (ACD) calculation using the IOL Master 500 (Version 5.0; Carl Zeiss Meditec Ltd, Jena, Germany). All tests were performed in the First Ophthalmology Department of Pomeranian Medical University in Szczecin, Poland.

### 2.3. Optical Coherence Tomography

All participants underwent spectral domain enhanced depth imaging-optical coherence tomography (EDI-OCT; Spectralis, Heidelberg Engineering, Germany) examination. The measurements were performed by an experienced technician (AG) after 30 minutes of rest and at the same time of the day (between 9.00 and 11.00 a.m.), after pupil dilation with a 1% tropicamide solution. In addition, patients were instructed not to smoke for 6 hours and not to drink any fluids for 1 hour before the examination. To obtain SD-OCT images of the macular region, a 25° × 25° volume acquisition protocol was used, achieving 49 cross-sectional B-scans. Choroidal segmentation was performed manually after the automated retinal layer segmentation software was disabled. The experienced retina specialist moved the reference lines of the built-in automated segmentation from the retinal boundaries to the choroidal boundaries. This method allowed us to use the automatic retinal thickness and volume map features of the built-in software. The automated software allowed choroidal volume calculations to be made in a manner similar to that for retinal volume analysis. Both CT and CV measurements were presented as the average of all points within the 9 Early Treatment Diabetic Retinopathy Study (ETDRS) subfields provided automatically by the Heidelberg Engineering software.

### 2.4. Medical Characteristics

The medical history recording, physical examination (including Doppler ultrasonography), and qualification for the CEA and surgical procedure were performed in The Department of Vascular Surgery at Pomeranian Medical University in Szczecin, Poland. Data regarding medical history, current drug use, smoking, and physical activity status were collected with a particular focus on the history of clinical cardiovascular diseases, including physician-diagnosed heart and vascular diseases. Furthermore, the actual arterial blood pressure (BP) was directly measured prior to ophthalmic examination in all subjects using a noninvasive blood pressure system with a manual aneroid manometer. The mean result from three measurements obtained with 5-minute resting intervals was calculated. From the obtained BP data, the systemic mean arterial pressure (MAP) was calculated as follows: MAP = diastolic BP + 1/3 (systolic BP − diastolic BP) mmHg. Furthermore, the following medical parameters were assessed in all patients: waist circumference (cm), waist/hip ratio (WHR), and body mass index (BMI) [weight (kg)/height (m)^2^]. Cumulative pack-years were calculated using the reported average number of cigarettes smoked per day and the number of years of smoking. Physical activity was expressed as the number of hours per week that the patient spent on moderate-intensity exercises. Haemodynamically significant internal carotid artery stenosis was defined as a greater than 70% reduction of the lumen diameter. The degree of ICAS was established with colour Doppler ultrasonography (CDU) using a Voluson 730 PRO, GE Medical System Device (Milwaukee, USA) with a multifrequency 7.5 MHz linear probe, according to the recommended guidelines in the literature.

### 2.5. Surgical Endarterectomy Procedure

The patients were preoperatively treated with oral acetylsalicylic acid (Aspirin, Bayer, Germany) at 75 mg/day for at least 10 days prior to the CEA. All patients underwent local anaesthesia to perform the CEA. Speech control monitoring and contralateral hand function were assessed throughout the procedure. The common, internal, and external carotid vessels were identified in all cases. Intravenous UF heparin (3000–5000 IU) was administered 5 minutes prior to cross-clamping all major vessels. After all vessels were cross-clamped, selective shunting was performed at the discretion of the operating surgeon if loss of consciousness was observed. The maximum carotid artery clamp time was 30 minutes. Endarterectomy without patch angioplasty was performed in the standard fashion with a 2.5× optical power magnification and Prolene 6/0 continuous sutures. Patch plasty was performed only if the native ICA had a diameter <5 mm in size. Postoperatively, all patients were administered antiplatelet therapy in combination with stringent blood pressure control and close observation in a high dependency unit for at least 24 hours. The antithrombotic therapy consisted of low molecular heparin (Clexane, Enoxaparinum natricum, Aventis, France) at 40 mg twice/day after the operation. Antiplatelet therapy was initiated the next day after surgery and then continued for lifetime. All patients were followed up both clinically and via colour doppler imaging after discharge. Any important postoperative complications of CEA (stroke, significant restenosis/occlusion, and cardiac disorders) excluded the patient from further participation in the trial. The study protocol was approved by the local ethics committee and was conducted in accordance with the guidelines of the Helsinki Declaration. All enrolled patients gave their written informed consent.

### 2.6. Statistical Analysis

The Shapiro–Wilk test was conducted to assess the normality of the distribution for continuous variables prior to the data analysis. Parameters were compared between independent groups with the Mann–Whitney test for quantitative variables. The Wilcoxon signed-rank test was used to assess whether the differences in parameters measured between the time points were significant. The Spearman rank correlation coefficient was used to evaluate associations between the measured variables. The significance level was determined as 0.05 in all statistical analyses.

## 3. Results

For the quantitative analysis of central choroid thickness and volume, the eyes were organized into two groups: the eyes on the side of the operated internal carotid artery (EIE—Eyes Ipsilateral to Endarterectomy) and the eyes on the side not subjected to surgery (ECE—Eyes Contralateral to Endarterectomy). A comparative analysis of the EDI-OCT data obtained before the surgery and the data recorded on the second day and in the third month after the CEA was performed.

Overall, 42 eyes of 21 consecutive patients (10 males, 11 females) with significant internal carotid artery stenosis that was subjected to unilateral endarterectomy were recruited to the study. Two patients were lost to follow-up due to weak compliance; thus, data from 19 participants were included for further analysis. The mean age of the subjects was 66.4 ± 8.2 years (ranging from 49 to 81 years). The median ICA stenosis degree on the side subjected to surgery was 81.5 ± 9.4% (70–99%), with a value of 29.5 ± 33.9% on the contralateral side. One patient (5.3%) had total occlusion of the ICA on the nonoperated side. Eleven participants (57.9%) suffered from hypertension, and 6 (31.6%) had ischaemic heart disease, including 4 persons (21.1%) with previous myocardial infarction. None of the participants had a history of stroke. The mean SBP was 129.3 ± 12.4 mmHg (ranging from 109 to 152 mmHg), the mean DBP was 80.5 ± 9.0 mmHg (61–97 mmHg), and the mean MAP was 96.7 ± 9.1 mmHg (83–109 mmHg). The mean WHR was 17 ± 0.07 (ranging from 0.82 to 1.03), and the mean BMI was 27.3 ± 2.8 kg/m^2^ (22.7–33.1 kg/m^2^). In the study group, 84.2% of participants were nicotine abusers. The number of pack-years of smoking varied from 0 to 40, with a mean value of 23. The mean AXL of the participants' eyes on the side subjected to the operation and on the opposite side was 23.32 ± 0.7 mm and 23.44 ± 0.65 mm, respectively, whereas the average ACD was 3.33 ± 0.65 versus 3.28 ± 0.49 mm, respectively. The mean IOP was 14.2 ± 3.23 mmHg (9.2–20.9 mmHg) and 14.0 ± 3.7 mmHg (9.5–20.5 mmHg) in the EIE and ECE groups, respectively. No significant changes in VA or in the intraocular pressure in either eye were detected at baseline or after the CEA treatment.

To estimate the potential effect of CEA on choroid parameters, we assessed choroidal thickness and volume on the day before the CEA, on the 2nd day, and 3 months after the surgical procedure. The detailed CT and CV values obtained in the EIE and ECE groups at baseline and after treatment are summarized in Tables 1 and 2. No significant differences in either CT or CV in the EIE before CEA or on the 2nd day after the surgical procedure were observed in any of the 9 ETDRS subfields. Importantly, we documented an increase in CT in the 3rd month after CEA compared with the baseline results in the analysed ETDRS subfields, except for the outer temporal and inferior choroidal ring areas. Likewise, when the volume values were analysed, a significant increase at 3 months after CEA compared to baseline was noted in total choroid volume, as well as in the ETDRS regions except for the central area, outer temporal, and superior choroidal ring areas.

Accordingly, when analysing the ECE group, we observed no significant differences in either CT or CV between the results recorded before and on the 2nd day after treatment in the 9 ETDRS subfields (Table 2). However, the CT was significantly lower in the inner superior ETDRS subfield on the 2nd day post-CEA compared with the initial data obtained before the surgical procedure (median: 319 versus 306 *μ*m, *p*=0.05). Likewise, no significant changes were identified in CT or CV data 3 months after CEA compared with the baseline results.

Interestingly, in the ECE group, when comparing the results obtained in the 2nd day and 3rd month after surgery, we found significantly increased central CT and CV. Additionally, CT was increased in both superior rings in the inner nasal and outer temporal segments. CV was significantly changed centrally in the superior segments of both rings and in the outer temporal area.

**Figure 1:**
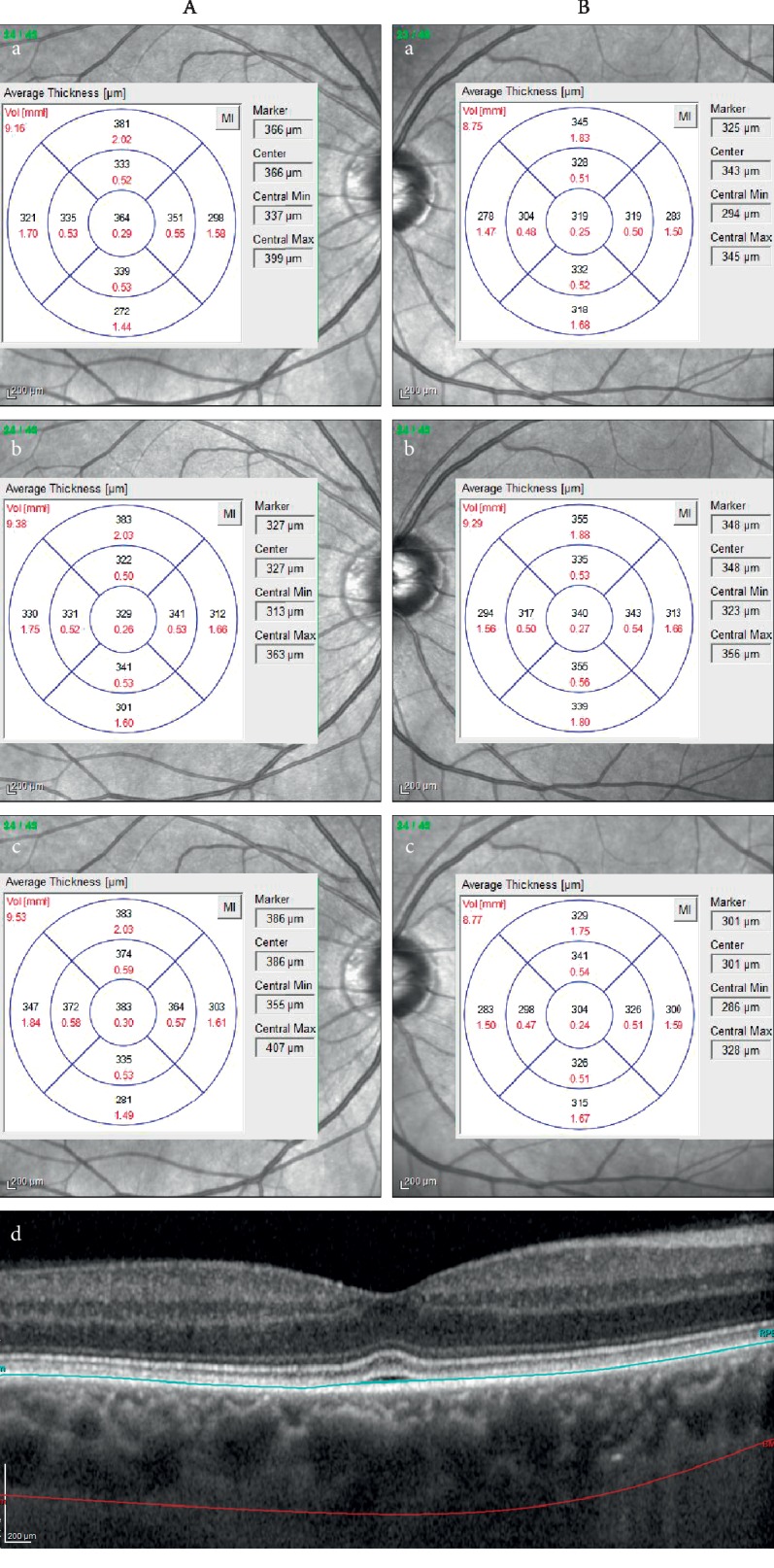
The measurements of choroidal volume (mm^3^) (marked in red) and thickness (*μ*m) (marked in black) presented for 9 Early Treatment Diabetic Retinopathy Study (ETDRS) areas of the eye ipsilateral (A) and contralateral (B) to the endarterectomy before (a), on the 2nd day (b), and on the 3rd month (c) after surgery. Enhanced depth imaging-optical coherence tomography (EDI-OCT) of macular region (d) with marked retinal pigment epithelium (blue line) and scleral-uveal junction (red line).

To extend the analysis of choroidal thickness within single EDTRS subfields, and to avoid certain inaccuracies resulting from the focal choroid thinning and uveoscleral border irregularities, we introduced total choroidal thickness (TCT) parameter, which was defined as the average thickness of all 9 EDTRS segments. In ICE group we observed an increase in TCT in the 3rd month after CEA compared with the baseline (*p*=0.04). Accordingly, when analysing the ECE group, we found significantly increased TCT when comparing the results obtained in the 2nd day and 3rd month after surgery (*p*=0.02).  Figure 1 shows representative analysis of the choroidal thickness and volume of a patient undergoing unilateral CEA.

To characterize the other factors that may influence the obtained results, we evaluated the potential association between the choroidal thickness and volume changes and several ophthalmic and clinical parameters (Tables 3 and 4). In the EIE group, the changes in choroidal volume and thickness in the central ring area between the data recorded before and in the 3rd month after the treatment were negatively correlated with the participant's age (Rs = −0.6; *p*=0.02 and Rs = −0.5; *p*=0.05, respectively). A similar correlation was found between the participant's age and the changes in total choroidal volume between the 3rd month and the 2nd day after the treatment (Rs = −0.61; *p*=0.02) in the ECE group. Additionally, in the EIE group, we found a negative correlation between ICA stenosis degree and changes in the central CT on the 2nd day after surgery compared to baseline (Rs = −0.5; *p*=0.05). In the light of the above, it is interesting to present data of the patient with 80% of ICA lumen reduction on the operated side and total carotid artery occlusion on the opposite side. He was a 69-year-old man with advanced central choroidal thinning in both eyes. Central choroidal thickness in the eye ipsilateral to CEA was 147 *μ*m at the baseline and changed to 167 *μ*m on the 2nd day and to 158 *μ*m in the 3rd month after surgery. In eye contralateral to CEA, the changes in central choroidal thickness were more pronounced than on ipsilateral side: 179 *μ*m at the baseline, 200 *μ*m on the 2nd day, and 216 *μ*m in the 3rd month after CEA. Such a symmetrical change of the choroidal thickness in both eyes after CEA would confirm the potential positive effect of unilateral CEA on the contralateral eye circulation in the choroid, most probably due to collateral circulation. In the ECE group, variation of the choroidal thickness between the 2nd day and 3rd month post-CEA in the central ring area showed a negative correlation with the smoking status of the patient (number of pack-years) (Rs = −0.5; *p*=0.04) and a positive correlation with the participant's physical activity status (Rs = 0.57; *p*=0.03 for total CV, Rs = 0.52; *p*=0.04 for central CV and Rs = 0.58; *p*=0.03 for central CT). Because some of the participants changed their smoking habits after surgical treatment of the carotids, we divided the group of patients with history of smoking into two subgroups: current smokers and former smokers. In ICE group, we observed that CV changes between 2nd day and 3rd month post-CEA were reduced in former smokers as compared to nonsmokers (median = 0.15 for smokers and 0.86 for nonsmokers; *p*=0.01). A similar association was found in ECE group (median = 0.16 for former smokers and 0.81 for nonsmokers; *p*=0.05). Moreover, choroidal volume changes measured 3 months after surgery compared to both baseline and the second day postsurgical results were positively correlated with diastolic blood pressure (Rs = 0.58, *p*=0.02; Rs = 0.52; *p*=0.04, respectively).

Of interest, we found significant positive correlations in choroidal parameters between EIE and ECE. CV variations on the 2nd day after surgery compared to baseline in EIE were positively correlated with CV changes in ECE (Rs = 0.66; *p*=0.007). Similar positive interconnections between both studied eyes were observed in choroidal volume variation results obtained in the 3rd month compared to the 2nd day after CEA (Rs = 0.76; *p*=0.001). This may indicate that compensatory vascular mechanisms from the collateral circulation originating in the brain-based circle of Willis are triggered after a unilaterally performed CEA.

## 4. Discussion

The blood supply to the choroid arises from the ophthalmic branch of the internal carotid artery (ICA) via the short ciliary arteries [17, 29]. Stenosis of the ICA results in the reduction of arterial pressure distal to the stenosis, including brain and ocular circulation. When the ICA is obstructed, the blood flow to the brain is compensated by the collateral circulation from the circle of Willis, which brings blood from the contralateral ICA through the communicating arteries and ipsilateral collateral channels between the terminal external carotid artery (ECA) branches and the terminal branches of the ophthalmic artery. In severe ICAS, the ophthalmic artery “stealing syndrome” may be present, leading to retrograde flow to the internal carotid artery and a reduction of blood flow in posterior ciliary arteries. This results in an insufficient blood perfusion of the choriocapillaris, alterations in the blood supply of the outer layers of retina, and changes to normal retinal function. Thus, there is a close relationship between choroid degenerative disease and insufficient blood perfusion of the choriocapillaris, which might be an important risk factor for the development of choroid degenerative disease [1, 30]. Initially, the ischaemic changes may be subtle and clinically inaccessible. Accordingly, we recently demonstrated that the retinal bioelectrical function is negatively affected by haemodynamically significant ICAS despite the absence of objective clinical signs and symptoms of ocular ischaemia [31]. The medical review of choroidal thickness in ICAS without ischaemic ocular syndrome gives conflicting results. Wang et al., based on a group of 219 patients with unilateral severe ICAS, found that the mean SFCT of the carotid stenosis group was significantly lower compared to that of normal eyes, probably in the mechanism of insufficient blood perfusion of the posterior ciliary arteries and choriocapillaris [22]. Similarly, Sayin et al. compared the eyes of severe ICAS patients with those of healthy volunteers and found a significantly thinner central choroid in the former group [29]. On the other hand, Akçay et al. found that the choroid was thicker in patients with unilateral significant ICA stenosis in a presumable mechanism of compensatory enlargement of the choriocapillaris vasculature to prevent retinal and choroidal ischaemia as a result of the diminished blood flow due to ICA stenosis [17]. Significantly, the young age of our study participants may provide some explanation.

Carotid endarterectomy is considered an effective method for reducing brain stroke risk in patients with severe ICAS [32, 33]. Numerous studies have shown its beneficial effect on blood flow in the ophthalmic artery, central retinal artery, posterior ciliary arteries, and chorioretinal circulation using Doppler ultrasonography and laser speckle flowgraphy [21, 22, 26]. Our study revealed significant differences in subfoveal choroidal thickness and volume 3 months after carotid endarterectomy compared to baseline in the EIS group. This is consistent with a previously published study by Lareyre et al. [28]. These data are in concordance with our previous investigation documenting that CEA for asymptomatic carotid artery stenosis is reproducibly associated with improvement of retinal function [23, 31]. In contrast, Rabina et al., based on a group of 8 patients with unilateral severe ICAS undergoing carotid endarterectomy, did not find any significant differences in SFCT between 6 and 14 months after surgery. The authors speculated that choroidal blood supplying the retina was sufficient despite the severe internal carotid stenosis in patients without ischaemic ocular syndrome signs due to preserved ophthalmic artery blood flow [27]. However, the study group was relatively small and predominantly male, and the participants were older. Importantly, all of them were hypertensive, which could have potentially affected their results [27].

Importantly, to our knowledge, this is the first study aimed at assessing the choroidal parameters in the early postoperative period on the 2nd day post-CEA. We found no significant changes in choroidal thickness or volume at this initial time point. The choroid consists mainly of blood vessels and the remaining part is extravascular stroma; both components can affect the thickness of the tissue [1–3]. The choroidal vessels are under autonomic control. Unilateral sympathetic nerve stimulation causes a substantial reduction in choroidal blood flow due to vasoconstriction [2, 3]. As a result of a carotid endarterectomy, a large volume of blood is suddenly delivered to the ocular circulation that may trigger mechanisms that counteract hyperaemia—stimulation of sympathetic nerves resulting in vasoconstriction in choroidal vessel, which is reflected in OCT as reduction of choroidal thickness. The thickness of the choroid is significantly influenced by the extravascular stroma, which accounts for approximately one-third of the entire tissue and, under certain conditions, allows the choroid to increase its thickness by 50% within an hour and quadruple in a few days. The aforementioned mechanisms may be affected by both long-standing ischaemia and sudden increased blood inflow after a successive endarterectomy. Thus, we can conclude that the choroidal thickness and volume increase observed in our study in the 3rd month was associated with the development of long-term adaptive mechanisms for improved blood inflow.

Interestingly, 3 months after surgery, we identified a significant improvement in choroidal thickness and volume in the eyes on the nonoperated side compared to the results obtained 2 days after surgery. Additionally, the analysis of changes in CV after surgery showed a strong positive correlation between the EIE and ECE groups. The CEA improves contralateral cerebral and ocular perfusion through the circle of Willis and ophthalmic arteries in the so-called redistribution phenomenon [22, 34]. Unilateral surgery for significant carotid artery stenosis may improve the ocular circulation on the opposite side. This is consistent with our previous observations documenting a significant improvement in retinal bioelectrical function in the eyes ipsilateral and contralateral to CEA [31].

Furthermore, our study revealed a positive effect of CEA on choroidal thickness and volume not only in the central part of the macula but also in the perifoveal areas. The changes were particularly visible in the superior and nasal segments, while in the inferior segments, there was no choroidal thickness or volume variations at any of the time points selected in this study. It is difficult to unequivocally explain this phenomenon. According to Hayreh, there is a large interindividual variation in choroidal vasculature, including the number and locations of short ciliary arteries or veins, as well as the size and position of watershed zones [35]. Kakiuchi et al. indicated that there was a smaller luminal area within the inferior perimacular region compared to the superior area in a healthy population. This is probably due to the influence of gravity on the direction of flow, which may lead to an increase in intravascular pressure within the choroid located in the superior parts [36]. Additionally, the final stage of disc cup closing is located within the lower segments, which is also a common location of a choroidal coloboma, which may indicate a weaker vascularization of this choroidal region [36, 37]. The above-mentioned causes may either make the inferior part of the perimacular region more susceptible to ischaemia or indicate different mechanisms of blood flow regulation. We are also aware of the impact of the relatively small number of participants in our study on the obtained results.

Because most of our participants were smokers, the effect of nicotine on choroidal thickness was taken into consideration. Smoking is known to be associated with anatomical and functional alterations in the microvasculature, leading to an increase in arterial wall thickness and stiffness as well as a peripheral vasoconstriction effect [38–41]. Smoking may lead to a decrease in retinal and choroidal blood flow, which might lead to structural changes in the choroid. Nicotine abuse is an important risk factor for many systemic and ocular vascular diseases, such as hypertensive retinopathy, AMD, and anterior ischaemic optic neuropathy (AION) [42, 43]. Several studies have shown a significant decrease in choroidal thickness in smokers that is probably related to vascular dysfunction caused by a decrease in nitric oxide (NO) bioavailability [41]. In the choroid, NO acts as a vasoactive factor released from cholinergic fibres of parasympathetic system innervated choroidal vessels [2, 3, 44]. Smoking-related altered NO metabolism may disturb the balance of both antagonistic components of the autonomic system that leads to vasoconstriction, strengthening the vascular resistance and, consequently, the choroidal thinning visualized on OCT [41]. In our study, variation of the choroidal thickness post-CEA showed a negative correlation with the smoking status of the patient. This may clearly reflect the pathological microvascular changes mediated by nicotine and smoking that lead to reduced choroidal vessel reactivity following the less pronounced changes in OCT parameters.

A similar negative correlation between a participant's age and choroidal thickness and volume variation after CEA was detected. This finding indicates that older patients showed a smaller increase in CT and CV compared with their younger counterparts. In contrast, central choroidal volume and thickness changes demonstrate a positive correlation with the participants' physical activity status. In contrast to smoking, regular physical training improves vascular function by increasing the production and bioavailability of antiatherogenic mediators as well as blood vessel remodelling, leading to an enlargement of the vessel lumen and a decrease of vascular resistance [45–47] The influence of physical exercises on eye circulation is complex but generally considered beneficial due to increasing ocular perfusion pressure [48]. Thus, as observed in our study, participants with higher physical activity levels presented greater increases in choroidal parameters after CEA.

Interestingly, our study revealed positive correlations between choroidal volume and thickness variations after CEA and diastolic blood pressures. Previous studies revealed a decrease in choroidal thickness in patients with chronic systemic arterial hypertension, which was most likely due to existing blood vessel sclerosis and vascular contraction caused by the high intravascular pressure in the choroid [49]. In contrast, in response to a sudden acute increase in blood pressure, an increase in CHT was reported [41]. The positive correlation between diastolic BP values and choroidal parameters observed in the current study may indicate alterations in blood vessel reactivity related to either chronic ischaemia due to ICAS or a sudden increase in blood flow to the choroidal vascular bed after surgery in hypertensive participants.

Altogether, correlation analysis showed a relationship between choroidal thickness and volume variation and cardiovascular risk factors. These factors altered microvessel reactivity in response to an increased ocular blood inflow after successful unilateral carotid endarterectomy for haemodynamically significant stenosis. Older age, smoking status, and low physical activity status are directly associated with accelerated atherosclerosis formation, which may explain the observed poorer vascular response after CEA treatment. Additionally, we noticed a negative correlation between degree of ICA stenosis and choroidal thickness changes on the second day after endarterectomy procedure indicating that participants with more severe stenosis are less likely to increase choroidal thickness. This may result from a greater stiffness of the atherosclerotic vessels or more pronounced vasoconstriction in response to the sudden increase in blood flow after CEA.

Our study has several limitations. A relatively small sample size may lead to statistical error, and some choroidal thickness and volume results did not reach statistical significance in the analyses of the 2nd day and 3rd month compared to baseline. A partial explanation may be the small number of participants. However, we consider our work to be valuable because of its prospective design. Furthermore, to our knowledge, this is the first research study using EDI-OCT to assess choroidal thickness after CEA in an early postoperative phase. We used a novel method for the evaluation of the choroid layer, with calculation of the CV in the whole 6 mm rings around the fovea and analysis in the 9 ETDRS fields. To minimize the risk of error (mismarks), we manually marked the scleral-uveal junction in all 31 available B-scans from each examination. The strength of the current study is the inclusion of choroid volume measurements for analysis. This parameter covers more than a single measurement at a particular point on the CT map based on a few marked distances and gives us important information on the whole 3D volume of the choroid layer. The analysis of CV provides more detailed and reliable information on choroidal parameters in eyes with circulatory alterations secondary to ICAS and subsequent CEA.

In summary, in patients with severe ICAS, we observed an increase in central choroidal thickness and total volume in the 3rd month after carotid endarterectomy compared to baseline. This effect was also visible in the perifoveal area. This rescue effect was noticed in both EIE and ECE eyes, but compared to baseline, the results reached statistical significance only in the eyes ipsilateral to CEA. Our observations indicate that EDI-OCT is a potentially valuable tool for evaluating the short- and long-term effectiveness of CEA in patients with haemodynamically significant ICAS. Choroidal thickness and volume fluctuations visualized by our group using the EDI-OCT technique enabled the observation of the processes of vascular tissue adaptation to variable blood flow conditions before and after CEA. The obtained results appear to be an additional point in the ongoing discussion of the credibility of CEA treatment in ICAS patients.

## Figures and Tables

**Table 1:** Choroidal thickness and volume values and variations before and after carotid artery endarterectomy in the EIE group.

Localization	EIE group					
	Baseline	2nd day after CEA	3rd month after CEA	*p* value		
				Baseline versus 2nd day	Baseline versus 3rd month	2nd day versus 3rd month
*Choroidal thickness (μm)*						
Central choroidal thickness	320 (73)	307 (84)	336 (85)	0.41	**0.03**	**0.02**
Total choroidal thickness	299 (72)	291 (77)	310 (77)	0.61	**0.04**	0.06

*Inner ring area*						
Temporal	300 (88)	290 (107.5)	318 (68)	0.52	**0.01**	**0.03**
Superior	290 (74)	298.5 (90)	319 (90)	0.96	**0.02**	**0.03**
Inferior	310 (121)	313 (135)	335 (87)	0.78	**0.05**	0.14
Nasal	298 (112)	315 (129)	325 (117)	0.42	**0.02**	**0.02**

*Outer ring area*						
Temporal	281 (102)	278 (87.5)	282 (62)	0.80	0.06	0.06
Superior	290 (107)	285 (120)	313 (113)	0.84	**0.04**	**0.02**
Inferior	272 (149)	280 (125)	257 (151)	0.32	0.11	0.95
Nasal	250 (158)	258.5 (148)	287 (143)	0.50	**0.03**	**0.01**

*Choroidal volume (mm)*						
Central choroidal volume	0.25 (0.05)	0.24 (0.07)	0.26 (0.06)	0.42	0.06	**0.01**
Total choroidal volume	8.07 (2.05)	8.1 (2.23)	8.52 (2.12)	0.86	**0.01**	**0.02**

*Inner ring area*						
Temporal	0.47 (0.13)	0.46 (0.17)	0.5 (0.11)	0.49	**0.02**	**0.03**
Superior	0.45 (0.12)	0.47 (0.15)	0.5 (0.14)	0.92	**0.01**	**0.03**
Inferior	0.49 (0.19)	0.49 (0.22)	0.53 (0.14)	0.75	**0.04**	0.07
Nasal	0.47 (0.17)	0.495 (0.2)	0.51 (0.18)	0.31	**0.03**	**0.02**

*Outer ring area*						
Temporal	1.37 (0.59)	1.47 (0.47)	1.5 (0.61)	0.29	0.06	0.24
Superior	1.53 (0.57)	1.52 (0.64)	1.66 (0.6)	0.85	0.06	**0.02**
Inferior	1.44 (0.79)	1.49 (0.67)	1.49 (0.7)	0.32	**0.01**	0.50
Nasal	1.33 (0.83)	1.37 (0.79)	1.52 (0.76)	0.43	**0.03**	**0.01**

CEA: carotid endarterectomy. Statistically significant results are indicated in bold (*p* ≤ 0.05).

**Table 2:** Choroidal thickness and volume values and variations before and after carotid artery endarterectomy in the ECE group.

Localization	ECE group					
	Baseline	2nd day after CEA	3rd month after CEA	*p* value		
				Baseline versus 2nd day	Baseline versus 3rd month	2nd day versus 3rd month
*Choroidal thickness (μm)*						
Central choroidal thickness	298.5 (95)	285 (103)	303.5 (84)	0.23	0.34	**0.02**
Total choroidal thickness	289 (72)	272 (65)	295 (65)	0.17	0.42	**0.02**

*Inner ring area*						
Temporal	302 (105)	284 (116)	309 (63)	0.12	0.82	0.09
Superior	319 (78)	274 (107)	306 (89.5)	0.05	0.55	**0.01**
Inferior	291.5 (86)	290 (101)	306.5 (74)	0.65	0.24	0.35
Nasal	290 (104)	272 (96)	295.5 (94.5)	0.18	0.36	**0.04**

*Outer ring area*						
Temporal	271.5 (115)	270 (85)	278.5 (64.5)	0.20	0.78	**0.03**
Superior	310 (126)	270 (104)	301.5 (105.5)	**0.05**	0.43	**0.03**
Inferior	259 (134)	252 (145)	261.5 (108.5)	0.43	0.09	0.06
Nasal	263 (120)	244 (97)	264.5 (105.5)	0.63	0.30	0.14

*Choroidal volume (mm)*						
Central choroidal volume	0.235 (0.07)	0.22 (0.08)	0.24 (0.07)	0.24	0.67	**0.04**
Total choroidal volume	7.87 (2.0)	7.53 (1.83)	8.07 (1.86)	0.19	0.21	**0.02**

*Inner ring area*						
Temporal	0.47 (0.16)	0.45 (0.18)	0.485 (0.1)	0.16	0.97	0.15
Superior	0.505 (0.13)	0.43 (0.14)	0.48 (0.15)	0.07	0.46	**0.01**
Inferior	0.455 (0.13)	0.46 (0.15)	0.48 (0.12)	0.58	0.33	0.25
Nasal	0.455 (0.16)	0.43 (0.15)	0.465 (0.15)	0.28	0.35	0.07

*Outer ring area*						
Temporal	1.44 (0.61)	1.43 (0.55)	1.475 (0.34)	0.11	0.82	**0.02**
Superior	1.64 (0.67)	1.43 (0.56)	1.595 (0.56)	0.06	0.44	**0.03**
Inferior	1.37 (0.71)	1.32 (0.77)	1.385 (0.58)	0.41	0.11	0.06
Nasal	1.39 (0.64)	1.29 (0.63)	1.4 (0.57)	0.49	0.39	0.14

CEA: carotid endarterectomy. Statistically significant results are indicated in bold (*p* ≤ 0.05).

**Table 3:** Correlation between choroidal thickness and volume and specific clinical features in the EIE group.

	Age		SBP		DBP		Smoking status		BMI		WHR		Physical activity		ICAS degree	
	Rs	*p*	Rs	*p*	Rs	*p*	Rs	*p*	Rs	*p*	Rs	*p*	Rs	*p*	Rs	*p*
TV 0/2	−0.13	0.62	0.19	0.48	0.29	0.3	0.25	0.35	−0.16	0.56	0.15	0.61	0.12	0.66	−0.17	0.51
TV 0/3	−0.25	0.34	0.21	0.43	0.41	0.11	−0.29	0.27	0.02	0.94	0.17	0.54	0.03	0.91	0.28	0.29
TV 2/3	−0.18	0.51	0.12	0.67	0.18	0.49	−0.39	0.13	0.21	0.44	0.12	0.7	0.04	0.89	0.04	0.88
CHV 0/2	−0.12	0.66	0.32	0.23	0.34	0.19	−0.02	0.93	−0.01	0.98	−0.34	0.21	0.21	0.4	−0.42	0.1
CHV 0/3	−**0.56**	**0.02**	0.27	0.3	0.42	0.09	0.04	0.87	0.04	0.89	−0.14	0.63	0.2	0.46	0.28	0.29
CHV 2/3	−0.37	0.16	0.21	0.43	0.28	0.29	0.03	0.92	0.3	0.3	−0.11	0.7	0.15	0.59	−0.18	0.5
CHT 0/2	−0.1	0.7	0.31	0.24	0.34	0.2	0.01	0.97	−0.17	0.53	0.02	0.94	0.29	0.28	−**0.5**	**0.05**
CHT 0/3	−**0.47**	**0.05**	0.23	0.37	0.34	0.18	0.17	0.52	0.03	0.9	−0.02	0.94	0.3	0.24	0.37	0.16
CHT 2/3	−0.28	0.29	0.12	0.67	0.1	0.72	−0.05	0.86	0.33	0.22	0.01	0.97	−0.05	0.86	−0.18	0.48

TV: total volume; CHV: central volume; CHT: central thickness; 0: baseline; 2: 2nd day after CEA; 3: 3rd month after CEA; 0/2: differences in measurements taken on the 2nd day after CEA and at baseline; 2/3: differences in measurements taken on the 2nd day and in the 3rd month after CEA; ICAS degree: degree of internal carotid artery stenosis on operated side; Rs: Spearman rank; *pp* value. Statistically significant results are indicated in bold (*p* ≤ 0.05).

**Table 4:** Correlation between choroidal thickness and volume and specific clinical features in the ECE group.

	Age		SBP		DBP		Smoking status		BMI		WHR		Physical activity		ICAS degree	
	Rs	*p*	Rs	*p*	Rs	*p*	Rs	*p*	Rs	*p*	Rs	*p*	Rs	*p*	Rs	*p*
TV 0/2	0.26	0.34	0.24	0.39	0.2	0.47	0.42	0.12	−0.1	0.73	0.14	0.63	−0.2	0.45	0.05	0.85
TV 0/3	−0.31	0.25	0.18	0.5	0.58	**0.02**	0.03	0.91	−0.29	0.27	−0.05	0.86	0.16	0.57	−0.14	0.60
TV 2/3	−0.61	**0.02**	−0.03	0.9	0.37	0.17	−0.48	0.07	−0.25	0.38	−0.11	0.73	0.56	**0.03**	−0.08	0.78
CHV 0/2	0.42	0.12	0.2	0.49	−0.1	0.71	0.5	0.06	−0.09	0.76	0.03	0.92	−0.18	0.53	0.04	0.89
CHV 0/3	−0.14	0.6	−0.12	0.65	0.2	0.46	0.11	0.68	−0.37	0.15	−0.01	0.95	0.18	0.5	−0.07	0.81
CHV 2/3	−0.44	0.1	0.00	0.99	0.52	**0.04**	−0.34	0.21	−0.26	0.36	0.05	0.86	0.52	**0.04**	−0.12	0.66
CHT 0/2	0.36	0.19	0.15	0.6	−0.02	0.94	0.44	0.1	0.02	0.93	0.00	1.00	−0.28	0.3	0.08	0.79
CHT 0/3	−0.16	0.55	−0.1	0.66	0.17	0.53	0.07	0.8	−0.29	0.27	−0.16	0.57	0.12	0.66	0.03	0.93
CHT 2/3	−0.37	0.17	−0.11	0.69	0.23	0.42	−0.53	**0.04**	−0.35	0.2	0.03	0.92	0.57	**0.03**	−0.11	0.67

TV: total volume; CV: central volume; CHT: central thickness; 0: baseline; 2: 2nd day after CEA; 3: 3rd day after CEA; 0/2: differences in measurements taken on the 2nd day after CEA and at baseline; 2/3: differences in measurements taken on the 2nd day and in the 3rd month after CEA; ICAS degree: degree of internal carotid artery stenosis on operated side; Rs: Spearman rank; *p*: *p* value. Statistically significant results are indicated in bold (*p* ≤ 0.05).

## Data Availability

The data used to support the findings of this study are available from the corresponding author upon request.
